# Factors affecting the immunogenicity of influenza vaccines in human

**DOI:** 10.1186/s12879-023-08158-3

**Published:** 2023-04-06

**Authors:** Qiuyi Xu, Hejiang Wei, Simin Wen, Jiamin Chen, Yuxuan Lei, Yanhui Cheng, Weijuan Huang, Dayan Wang, Yuelong Shu

**Affiliations:** 1 School of Public Health (Shenzhen), Sun Yat-sen University-Shenzhen Campus, Shenzhen, Guangdong China; 2 National Institute for Viral Disease Control and Prevention, Chinese Center for Disease Control and Prevention, Beijing, China; 3 Clinical Research Center, Guangzhou First People’s Hospital, the Second Affiliated Hospital of South China, University of Technology, Guangzhou, Guangdong China; 4 Institute of Pathogen Biology, Chinese Academy of Medical Sciences & Peking Union Medical School, Beijing, China

**Keywords:** Influenza, Influenza vaccine, Vaccine effectiveness, Immune response, Immunogenicity

## Abstract

**Background:**

The influenza viruses pose a threat to human health and medical services, and vaccination is an important way to prevent infection. However, the effectiveness of influenza vaccines is affected by various aspects. This study aimed to explore factors related to the immune response to influenza vaccines.

**Methods:**

The study was conducted from September 2019 to September 2021, and a total of 593 volunteers were recruited from the Center for Disease Control and Prevention in 3 provinces in China. The hemagglutination inhibition assay was used to measure antibody levels. The Chi-square test, multivariable logistic regression analysis, and sum-rank test were used to analyze the factors associated with influenza vaccine immune response.

**Results:**

The Chi-square test showed that seroconversion rates and response rate were associated with age group, vaccination history, chronic conditions, the frequency of colds, and region (*P* < 0.05). The multivariable logistic regression analysis showed that age was an important factor that affected participants’ seroconversion rates for A/H1N1, A/H3N2, B/Victoria, and response status (18–64 vs. ≤5: OR = 2.77, *P* < 0.001; ≥65 vs. ≤5: OR = 0.38, *P* = 0.01; 18–64 vs. ≤5: OR = 2.64, *P* = 0.03). Vaccination history was also an affecting factor for A/H1N1, B/Victoria, and response status (yes vs. no: OR = 0.4 / 0.44 / 0.25, *P* < 0.001). The frequency of colds and chronic conditions were also affecting factors for participants’ seroconversion rates and response levels to different degrees. The sum-rank test showed that the fold changes for A/H1N1, B/Victoria, and B/Yamagata were associated with age group and vaccination history (*P* < 0.01). The fold changes for A/H3N2 were associated with the frequency of colds (*P* < 0.05), and those for B/Victoria were associated with gender and chronic conditions (*P* < 0.05).

**Conclusions:**

Vaccination history, age, health condition, and frequency of colds were important factors affecting the seroconversion rate of the influenza vaccine in human. There is a need for developing optimized vaccination strategies for vulnerable groups to improve the efficacy of influenza vaccines in human.

## Introduction

The influenza viruses are enveloped negative-sense single-strand RNA viruses with a segmented genome, including types A, B, C, and D. [1] Influenza A and influenza B viruses cause seasonal epidemics annually. [2] Among them, the major circulating strains include influenza A H1N1, A H3N2, B/Victoria and B/Yamagata lineages. [1] There are an estimated 1 billion influenza cases worldwide each year, including 3 to 5 million severe cases and 290, 000 to 650, 000 deaths, [3] of which pose a threat to human health and medical services.

Vaccination is the most effective way to reduce human influenza disease burden. [2] The risk of seeking treatment will decrease by 40–60% if influenza vaccine viruses match circulating viruses. [4] However, such protection effectiveness may be lower for some reasons, especially when the vaccine strains are mismatched with circulating viruses. [5] The immunogenicity of the vaccine is also one of the most important factors influencing vaccine effectiveness. [6] Previous studies indicate that the immunogenicity of the vaccine can be affected by repeated vaccination; [7] vaccine factors, such as vaccine types, dosage, and delivery mode of vaccine; [8–10] and host factors, such as age, gender, and health conditions. [6, 11]

In this study, 557 volunteers were recruited from three provinces in China and then vaccinated with the influenza vaccines to explore the factors associated with the vaccine immunogenicity. Several factors associated with responsiveness to influenza vaccination were identified. The results may provide supporting data for identifying influenza vaccination low responders and optimizing the vaccination strategies, thereby improving the effectiveness of the influenza vaccine in human.

## Materials and methods

### Participants and data Collection

Based on our previous research, [12] 593 volunteers were recruited by the staff of the Center for Disease Control (CDC) and Prevention of Yunnan Province, Shaanxi Province, and Xinjiang Uygur Autonomous Region from September 2019 to September 2021. We enrolled volunteers who were: (1) Han Chinese, (2) and had not already received the northern hemisphere formulation of influenza vaccine for the corresponding year. Volunteers were excluded if they: (1) reported medical conditions not suitable to receive influenza vaccines such as any allergic reaction to egg protein or previous dose of influenza vaccine; (2) reported medical conditions not suitable for intramuscular injection or venous blood collection such as the use of anticoagulant medication. As a result, 36 volunteers were excluded, and 557 were eligible for further research.

**Fig. 1:**
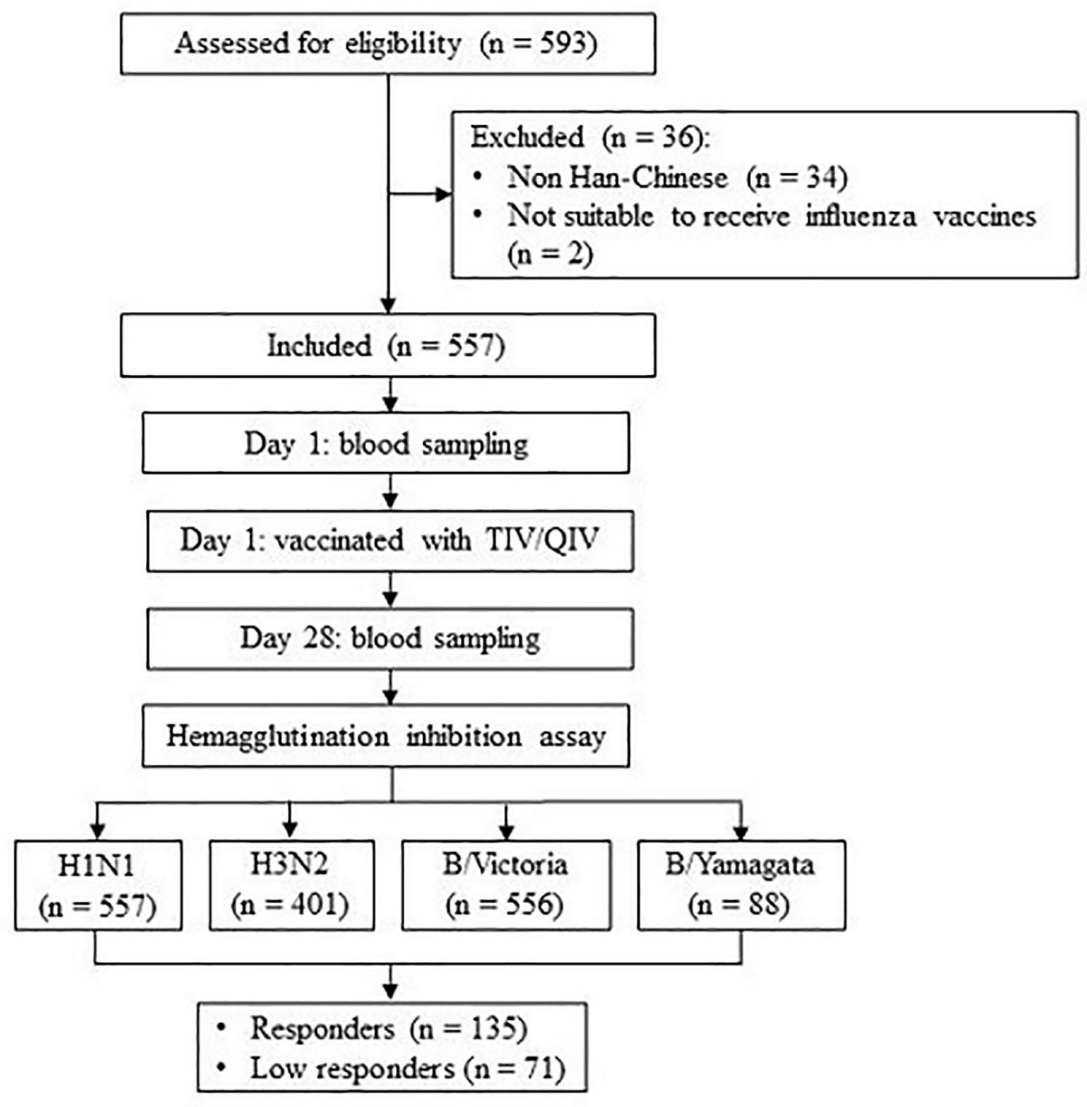
Flow Chart

The following information of volunteers was collected by the staff of CDC via questionnaire: gender, age, height, weight, region, vaccine type, frequency of colds, vaccination history, smoking and alcohol consumption, and health conditions. A total of 10 ml peripheral venous blood was collected by the staff of CDC before (day 1) and 28 days after the vaccination (day 28). The serum was isolated after blood samples setting for 4 h and stored in a − 80 °C ultra-low temperature freezer (Thermo Fisher Scientific, USA). The flow chart was described in Fig. 1.

All volunteers signed informed consent. The study was approved by the Ethics Review Committee of the National Institute for Viral Disease Control and Prevention, Chinese Center for Disease Control and Prevention (NIVDC, assurance number, 202023).

### Vaccines

All volunteers received trivalent inactivated vaccine (TIV) or quadrivalent inactivated vaccine (QIV) by intramuscular injection. The vaccines strains consisted of the Northern Hemisphere vaccine components recommended by the World Health Organization (WHO), and details were shown in Table 1. The TIV contained 15 µg hemagglutination (HA) for 3 strains, including A H1N1, A H3N2, and either B/Victoria or B/Yamagata lineage, and was provided by Shenzhen Sanofi Pasteur Biological Products Co., Ltd. The QIV contained 15 µg HA for 4 strains above and was provided by HUALAN BIOLOGICAL ENGINEERING, INC. Volunteers aged 5 years or older got 1 dose of vaccine containing 15 µg HA for each strain on day 1, and volunteers younger than 5 years got 2 doses of vaccine containing 7.5 µg HA for each strain with an interval of 30 days. All the vaccines were free for volunteers.

**Table 1:** Vaccine strains recommended by WHO for northern hemisphere from 2019–2021 and vaccination details

Influenza season	Vaccine strains	Vaccine type	Vaccinated number
2019–2020	A/Brisbane/02/2018 (H1N1)pdm09-like virus	TIV	179
	A/Kansas/14/2017 (H3N2)-like virus		
	B/Colorado/06/2017-like virus (B/Victoria/2/87 lineage)		
	B/Phuket/3073/2013-like virus (B/Yamagata/16/88 lineage)		
2020–2021	A/Guangdong-Maonan/SWL1536/2019 (H1N1)pdm09-like virus	TIV	214
	A/Hong Kong/2671/2019 (H3N2)-like virus		
	B/Washington/02/2019 (B/Victoria lineage)-like virus		
	B/Phuket/3073/2013 (B/Yamagata lineage)-like virus.		
2021–2022	A/Victoria/2570/2019 (H1N1)pdm09-like virus	QIV	164
	A/Cambodia/e0826360/2020 (H3N2)-like virus		
	B/Washington/02/2019 (B/Victoria lineage)-like virus		
	B/Phuket/3073/2013 (B/Yamagata lineage)-like virus		

### Hemagglutination inhibition assay

Hemagglutination inhibition (HAI) assay was used to measure the serum antibody titers against vaccine strains. The influenza virus strains used in the study were provided by the Chinese National Influenza Center. They were cultured by specified pathogen-free (SPF) chicken embryos (Xin Xing Da Hua Nong Poultry and Egg Co., LTD, China). 1% red blood cells from turkey (Guangzhou Hongquan Biotechnology Co., LTD, HQ80085-2, China) were used for influenza A/H1N1 and B HAI assays, and those from guinea pigs (Guangzhou Hongquan Biotechnology Co., LTD, HQ80077-4, China) were used for influenza A/H3N2 HAI assays. Before the assay, the serum was mixed with receptor-destroying enzyme (RDE) (Denka Seiken Co., Ltd., 340016, Japan) in a ratio of 1:3 in order to remove the non-specific inhibitor. The mixture was bathed in a 37 °C water bath (Jiangsu Keyi Instrument Co., LTD, China) for 16–18 h and then in a 56 °C water bath (Jiangsu Keyi Instrument Co., LTD, China) for 30 min for inactivation. The mixture was diluted to a final dilution of 1:5 in phosphate-buffered saline (Biosharp, BL302A, China) and then serially diluted 2-fold down a U-bottom 96-well plate for A/H3N2 (V-bottom 96-well plate for A/H1N1, B/Victoria, and B/Yamagata). 25 µl live virus containing 4 HAU was added to each well for a final 50 µl/well volume. After being incubated for 15–30 min at room temperature, 50 µl of 1% red blood cell was added to each well. After incubating for 30–60 min, the lowest dilution ratio leading to complete hemagglutination inhibition was recorded as the antibody titer. Operations involving the influenza virus were conducted in the Biosafety Level II laboratory.

The seroconversion rate referred to the proportion of participants showing a four-fold increase in post-immunization compared with pre-immunization titers, or titers < 10 pre-immunization to at least 40 post-immunization. [13] The seroprotection rate referred to the proportion of participants with a post-vaccination titer ≥ 40.^13^ The logarithm of antibody titer post-vaccination was divided by the logarithm pre-vaccination to obtain the fold change for antibody titer. Responders were defined as participants whose HAI titers achieved seroconversion to all influenza vaccine strains. Low responders were those whose HAI titers failed to achieve seroconversion to all influenza vaccine strains. The response rate referred to the proportion of responders to the sum of responders and low responders.

### Statistical analysis

Descriptive statistics were used to describe the participants’ characteristics, including mean (M), standard deviation (SD), frequency, and percentage. According to the HAI assay, the seroconversion status for each vaccine strain was categorized into “seroconversion” and “non - seroconversion”. For further analysis, the participants were categorized into “responders” and “low responders”. Independent variables include gender, age group, region, vaccine type, frequency of colds, vaccination history, body mass index (BMI) group, smoking, and alcohol consumption, and health conditions. The Chi-square test was used to compare the proportions of participants among different groups first. Associations of independent variables with the seroconversion were analyzed by multivariable logistic regression models adjusted for gender and age. Mann-Whitney U test or Kruskal-Wallis H test was used to explore the associations of independent variables with the fold changes since the fold changes among different groups were not normally distributed according to the results of the Kolmogorov-Smirnov test and Shapiro-Wilk test. Statistical significance was defined as *P* < 0.05. SPSS Statistics for Windows, Version 25.0 was used for statistical analysis. GraphPad Prism for Windows, Version 9.3.1 was used for processing graphs.

## Results

### Characteristics of participants

A total of 593 volunteers were recruited from 3 provinces of China, and 557 volunteers were included in the final analysis after 36 were excluded, whose details were shown in Table 2. The participants ranged from 2 to 89 years old, with a mean age of 49.4 ± 28. 6 years, and 60.7% (n = 338) were female. 42.9% (n = 239) were from Yunnan Province, 39.9% (n = 222) of participants were from Shaanxi Province, and 17.2% (n = 96) were from Xinjiang Uygur Autonomous Region. A total of 179 (32.6%) reported having a vaccination history, while 370 (67.4%) hadn’t been vaccinated. In the 2019–2020 and 2020–2021 season, 179 (32.2%) and 214 (38.4%) participants were vaccinated with TIV, respectively. In the 2021–2022 season, 164 (29.4%) were vaccinated with QIV. 34.0% (n = 188) of participants’ BMI was between 18.5 and 23.9, 30.7% (n = 170) was < 18.5, 23.9% (n = 132) was between 24 and 27.9, and only 11.4% (n = 63) was ≥ 28. When asked about the frequency of colds, 67.1% (n = 308) of participants reported 2–3, 17.2% (n = 79) reported 4–6, followed by ≤ 1 (11.3%, n = 52), and only 4.4% (n = 20) reported >6. Most participants reported not smoking (85.8%, n = 442) and not drinking (81.9%, n = 420). 76.6% (n = 422) of participants didn’t report chronic diseases. The seroconversion rates for A/H1N1, A/H3N2, B/Victoria, and B/Yamagata were 57.1%, 58.6%, 57.0%, and 52.3%, respectively. There were 135 (24.2%) responders and 71 (12.7%) low responders. The fold changes for A/H1N1, A/H3N2, B/Victoria, and B/Yamagata were 1.47(1.00-2.72), 1.60(1.23–2.20), 1.60(1.00-2.72), and 1.38(1.00-1.92), respectively.

**Table 2:** Characteristics of Participants in Different Groups (n = 557)

Variable	Category	Frequency/Mean/Median	Percentage/ Standard Deviation/ (P_25_, P_75_)
Gender	Male	219	39.3%
	Female	338	60.7%
Age Group	≤ 5	141	25.3%
	6–17	36	6.5%
	18–64	226	40.6%
	≥ 65	154	27.6%
Region	Yunnan	239	42.9%
	Xinjiang	96	17.2%
	Shaanxi	222	39.9%
Frequency of Colds	≤ 1	52	11.3%
	2–3	308	67.1%
	4–6	79	17.2%
	>6	20	4.4%
Vaccination History	No	370	67.4%
	Yes	179	32.6%
BMI Group	< 18.5	170	30.7%
	18.5–23.9	188	34.0%
	24-27.9	132	23.9%
	≥ 28	63	11.4%
Smoking	No	442	85.8%
	Yes	73	14.2%
Alcohol Drinking	No	420	81.9%
	Yes	93	18.1%
Chronic Diseases	No	422	76.6%
	Yes	129	23.4%
Vaccine Type	TIV	393	70.6%
	QIV	164	29.4%
Seroconversion for A/H1N1	No	239	42.9%
	Yes	318	57.1%
Seroconversion for A/H3N2	No	166	41.4%
	Yes	235	58.6%
Seroconversion for B/Victoria	No	239	43.0%
	Yes	317	57.0%
Seroconversion for B/Yamagata	No	42	47.7%
	Yes	46	52.3%
Response status	Low Responder	71	12.7%
	Responder	135	24.2%
	Neither	351	63.1%
Age	-	40.4	28.6
Height/cm	-	144.3	28.0
Weight/kg	-	49.1	23.0
BMI	-	21.5	4.8
The fold change for A/H1N1	-	1.47	1.00-2.72
The fold change for A/H3N2	-	1.60	1.23–2.20
The fold change for B/Victoria	-	1.60	1.00-2.72
The fold change for B/Yamagata	-	1.38	1.00-1.92

BMI: body mass index; TIV: trivalent inactivated vaccine; QIV: quadrivalent inactivated vaccine; P_25_: Lower quartile; P_75_: Upper quartile

Frequency of Colds: The frequency of common colds caused by adenovirus, parainfluenza virus, rhinovirus, respiratory syncytial virus (RSV), enterovirus, and coronavirus, etc. with mild upper respiratory syndromes per year; Vaccination history: Influenza vaccination history in the past 5 years. BMI Group: The grouping critiria was WS/T 428–2013. Smoking: Smoking in the past 5 years. Alcohol Drinking: Alcohol drinking in the past 5 years. Chronic Diseases: Diagnosed or treated for chronic medical condition during the past 5 years

### Associations between independent variables and responsiveness to influenza vaccination

The results of the Chi-square test were shown in Table 3. Seroconversion rates for A/H1N1, B/Victoria, B/Yamagata, and response rate were associated with the age group (*P* < 0.05), and participants aged between 6 and 18 years old had better responsiveness to the influenza vaccines. Seroconversion rates for A/H1N1, A/H3N2, and B/Victoria were associated with the region (*P* < 0.05). In addition, the seroconversion rate for A/H3N2 and response rate were related to the frequency of colds (*P* < 0.05). Participants from Xinjiang Uygur Autonomous Region or with a lower frequency of colds had worse responsiveness. Besides, seroconversion rates for A/H1N1, B/Victoria, and response rate were associated with repeated vaccination (*P* < 0.05). Furthermore, seroconversion rates for B/Victoria, and B/Yamagata were associated with chronic conditions (*P* < 0.05). Those without a vaccination history or chronic conditions had better responsiveness to the influenza vaccine.

**Table 3:** The Seroconversion Rate and Response Rate among Different Groups (n = 557)

Variable	Category	A/H1N1			A/H3N2			B/Victoria			B/Yamagata			Response Status		
		The seroconv-ersion rate	χ^2^	*P*	The seroconv-ersion rate	χ^2^	*P*	The seroconv-ersion rate	χ^2^	*P*	The seroconv-ersion rate	χ^2^	*P*	The Response rate	χ^2^	*P*
Age Group	≤ 5	51.1%	13.27	0.004	66.0%	4.03	0.26	53.2%	11.27	0.01	42.9%	8.50	0.04	60.0%	8.68	0.03
	6–17	72.2%			63.3%			71.4%			57.1%			88.2%		
	18–64	63.7%			56.7%			62.8%			67.6%			70.8%		
	≥ 65	49.4%			53.3%			48.7%			30.4%			53.3%		
Region	Yunnan	61.1%	29.40	< 0.001	80.7%	85.21	< 0.001	62.3%	17.48	< 0.001	-	-	-	71.0%	2.57	0.28
	Xinjiang	32.3%			86.5%			37.9			-			64.9%		
	Shaanxi	63.5%			38.3%			59.5%			52.3%			59.2%		
Frequency of Colds	≤ 1	63.5%	3.62	0.31	9.1%	34.34	< 0.001	51.9%	6.23	0.10	50.0%	2.88	0.41	50.0%	5.66	0.13
	2–3	63.3%			50.7%			64.6%			59.1%			71.7%		
	4–6	54.4%			77.5%			51.9%			30.0%			54.5%		
	>6	75.0%			55.6%			60.0%			50.0%			60.0%		
Vaccination History	No	64.3%	27.23	< 0.001	58.1%	0.13	0.72	64.8%	28.29	< 0.001	56.9%	1.72	0.19	72.7%	14.62	< 0.001
	Yes	40.8%			60.0%			40.8%			41.9%			43.1%		
Chronic Diseases	No	60.3%	1.10	0.29	53.9%	0.07	0.79	63.6%	11.48	0.001	64.1%	5.19	0.02	67.8%	1.18	0.28
	Yes	54.7%			55.7%			45.3%			33.3%			58.1%		

The results of the multivariable logistic regression analysis are presented in Table 4. Age (18–64 vs. ≤5: OR = 2.77, *P* < 0.001) and repeated vaccination (yes vs. no: OR = 0.4, *P* < 0.001) were important factors that affected participants’ seroconversion rates against A/H1N1. As for A/H3N2, age (≥ 65 vs. ≤5: OR = 0.38, *P* < 0.05) and frequency of colds (4–6 vs. ≤1: OR = 10.71, *P* < 0.05) affected the seroconversion rate. In the case of B/Victoria, age (18–64 vs. ≤5: OR = 2.45, *P* < 0.001), vaccination history (yes vs. no: OR = 0.44, *P* < 0.001), and chronic conditions (yes vs. no: OR = 0.48, *P* < 0.01) affected the seroconversion rate to influenza vaccines. Regarding B/Yamagata, chronic conditions (yes vs. no: OR = 0.18, *P* < 0.05) also affected the seroconversion rate. Similarly, age (18–64 vs. ≤5: OR = 2.64, *P* < 0.05) and vaccination history (yes vs. no: OR = 0.25, *P* < 0.001) were factors influencing participants’ response status to vaccine strains.

**Table 4:** Multivariable Logistic Regression Analysis of Responsiveness to Influenza Vaccine

Variable	Category	A/H1N1		A/H3N2		B/Victoria		B/Yamagata		Response Status	
		OR(95%CI)	*P*	OR(95%CI)	*P*	OR(95%CI)	*P*	OR(95%CI)	*P*	OR(95%CI)	*P*
Age Group	≤ 5	1.00	-	1.00	-	1.00	-	1.00	-	1.00	-
	6–17	2.83(0.98–8.12)	0.05	1.51(0.38–6.08)	0.56	2.30(0.87–6.11)	0.09	1.91(0.36–10.12)	0.45	4.31(0.43–43.07)	0.21
	18–64	2.77(1.68–4.57)	< 0.001	0.80(0.42–1.54)	0.51	2.45(1.51-4.0)	< 0.001	2.45(0.58–10.28)	0.22	2.64(1.08–6.46)	0.03
	≥ 65	1.32(0.76–2.28)	0.32	0.38(0.18–0.81)	0.01	1.46(0.86–2.47)	0.16	0.36(0.07–1.89)	0.23	1.16(0.42–3.25)	0.78
Frequency of Colds	≤ 1	1.00	-	1.00	-	1.00	-	1.00	-	1.00	-
	2–3	1.17(0.56–2.46)	0.68	4.00(0.75–21.58)	0.11	1.28(0.63–2.60)	0.50	1.39(0.45–4.33)	0.57	1.38(0.33–5.69)	0.66
	4–6	0.74(0.31–1.78)	0.50	10.71(1.68–68.05)	0.01	0.70(0.30–1.63)	0.41	0.30(0.04–2.14)	0.23	0.50(0.10–2.50)	0.40
	>6	2.15(0.63–7.36)	0.22	7.11(0.75–67.68)	0.09	1.16(0.38–3.54)	0.79	1.06(0.11–9.89)	0.96	0.80(0.08–8.08)	0.85
Vaccination History	No	1.00	-	1.00	-	1.00	-	1.00	-	1.00	-
	Yes	0.40(0.26,0.62)	< 0.001	0.73(0.38–1.41)	0.35	0.44(0.29–0.67)	< 0.001	0.87(0.30–2.52)	0.79	0.25(0.11–0.56)	< 0.001
Chronic Diseases	No	1.00	-	1.00	-	1.00	-	1.00	-	1.00	-
	Yes	0.83(0.51–1.34)	0.44	0.77(0.92 − 0.51)	0.77	0.48(0.30–0.78)	0.003	0.18(0.04–0.76)	0.02	0.66(0.28–1.57)	0.66

OR: odds ratio; CI: confidence interval

### Associations between independent variables and antibody fold change after vaccination

**Fig. 2:**
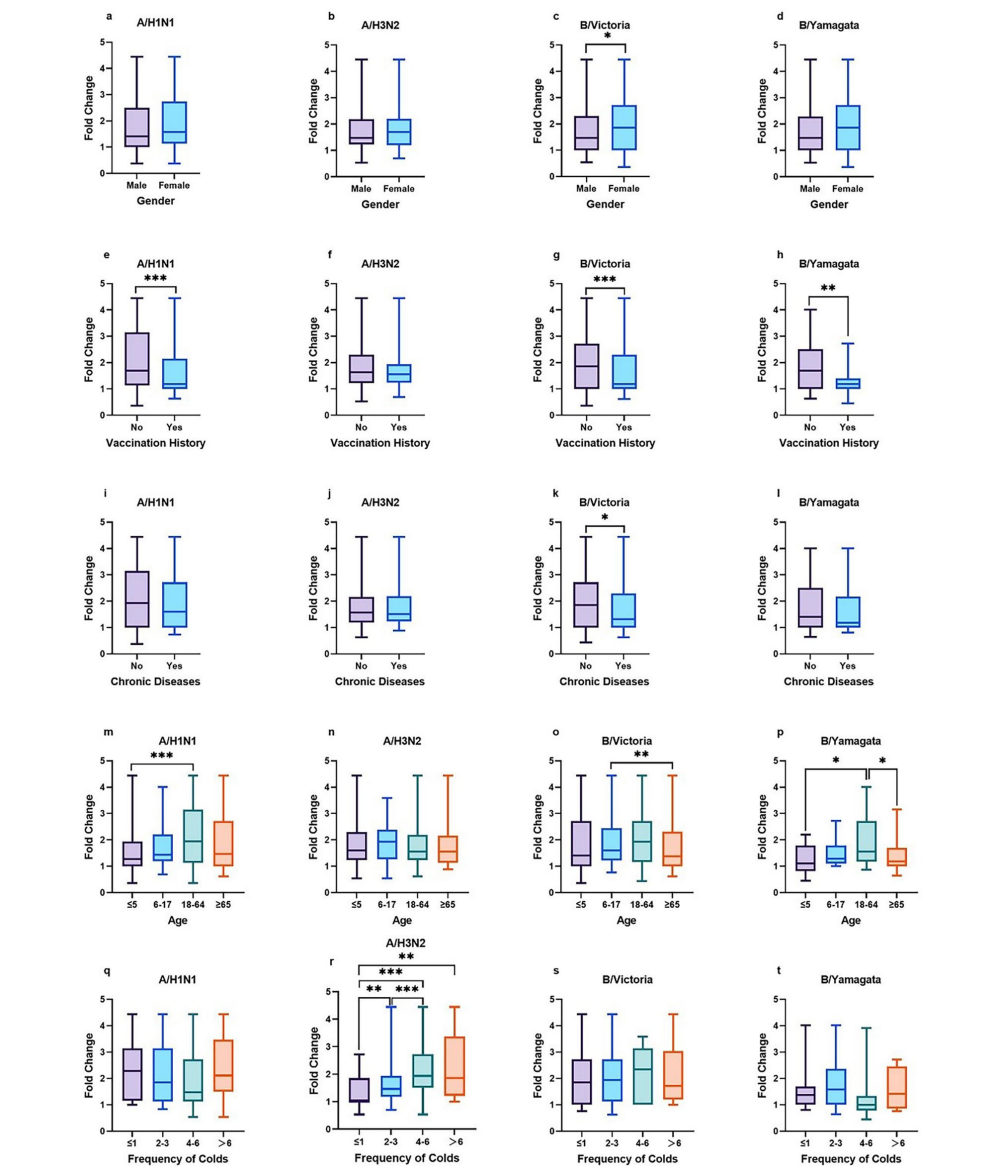
**Rank-Sum Test of Antibody Fold Change. Comparison of antibody fold changes against 4 vaccine strains. (a ~ d)** Comparison of antibody fold changes between male and female participants against 4 vaccine strains. **(e ~ h)** Comparison of antibody fold changes between participants with and without vaccination history against 4 vaccine strains. **(i ~ l)** Comparison of antibody fold changes between participants with and without chronic diseases against 4 vaccine strains. **(m ~ p)** Comparison of antibody fold changes among participants in 4 age groups against 4 vaccine strains. **(q ~ t)** Comparison of antibody fold changes among participants with 4 types of frequency of colds against 4 vaccine strains

As shown in Table 5; Fig. 2, the fold changes for A/H1N1, B/Victoria, and B/Yamagata were associated with age group (*P* < 0.05) and vaccination history (*P* < 0.05). Consistent with the results above, participants aged from 18 to 65 or without vaccination history had higher antibody folds. The fold changes for B/Victoria were associated with gender (*P* < 0.05) and chronic conditions (*P* < 0.05), and those who were female or without chronic diseases had higher antibody folds. Besides, the fold changes for A/H3N2 were associated with the frequency of colds (*P* < 0.05), and participants with a higher frequency of colds had higher folds.

**Table 5:** Rank-Sum Test of Antibody Fold Change

Variable	Category	A/H1N1		A/H3N2		B/Victoria		B/Yamagata	
		M(P_25_,P_75_)	*P*	M(P_25_,P_75_)	*P*	M(P_25_,P_75_)	*P*	M(P_25_,P_75_)	*P*
Gender	Male	1.86(1.00-2.72)	0.18	1.43(1.16–1.90)	0.33	1.63(1.00-2.72)	0.03	1.32(1.00-1.75)	0.21
	Female	1.88(1.16–3.15)		1.62(1.16–2.20)		2.29(1.12–2.74)		1.38(1.00-2.39)	
Age Group	≤ 5	1.27(1.00-2.14)	< 0.001	1.56(1.19–1.94)	0.68	1.39(1.00-2.72)	0.003	1.00(0.80–1.72)	0.04
	6–17	1.94(1.28–2.42)		1.37(1.00-1.90)		2.11(1.00-2.74)		1.29(1.10–1.79)	
	18–64	2.34(1.27–3.26)		1.69(1.23–2.20)		2.29(1.19–3.15)		1.60(1.19–2.72)	
	≥ 65	1.86(1.00-3.15)		1.30(1.00-1.86)		1.65(1.00-2.72)		1.17(1.00-1.82)	
Frequency of Colds	≤ 1	2.29(1.16–3.15)	0.3	1.00(1.00-1.54)	< 0.001	1.86(1.00-2.72)	0.58	1.38(1.00-1.69)	0.09
	2–3	1.86(1.14–3.15)		1.47(1.19–1.94)		1.94(1.12–2.72)		1.56(1.00-2.39)	
	4–6	1.47(1.07–2.72)		1.94(1.47–2.74)		1.47(1.00-2.94)		1.00(0.69–1.28)	
	>6	2.12(1.50–3.48)		1.86(1.22–3.37)		1.71(1.20–3.05)		1.43(0.86–2.47)	
Vaccination History	No	2.16(1.14–3.15)	< 0.001	1.56(1.18–2.20)	0.20	2.29(1.00-3.15)	< 0.001	1.69(1.00-2.51)	0.005
	Yes	1.38(1.09–2.29)		1.43(1.09–1.90)		1.29(1.00-2.29)		1.19(1.00-1.41)	
Chronic Diseases	No	2.25(1.14–3.15)	0.20	1.60(1.19–2.20)	0.98	1.94(1.16–2.74)	0.02	1.49(1.05–2.67)	0.34
	Yes	1.86(1.16–3.05)		1.53(1.23–2.36)		1.63(1.14–2.67)		1.31(1.00-2.02)	

M: median; P_25_: lower quartile; P_75_: upper quartile

## Discussion

Immunogenicity response to the vaccine is a complicated process, which is affected by many factors, including vaccine and host factors. [6, 14] In this study, we identified several factors associated with the responsiveness to the influenza vaccine. The Chi-square test, multivariable logistic regression analysis, and sum-rank test showed that vaccination history, chronic condition, age, and frequency of colds were important factors affecting immunogenicity responsiveness to the influenza vaccine. These results will help optimize influenza vaccination strategies and improve the effectiveness of the vaccine in humans.

In this study, participants with a vaccination history presented worse immunogenicity responsiveness to the influenza vaccines, indicating that continuous annual vaccination may lead to reduced vaccine efficacy, consistent with previous studies. A study evaluating the immunogenicity of trivalent inactivated influenza vaccine showed that the odds of seroconversion were strongly related to the baseline antibody titer, and the odds of seroconversion decreased dramatically when the baseline antibody level was higher. [15] Participants who received influenza vaccines in the previous year had significantly lower odds of seroconversion than those who did not. [15] One possible explanation was that repeated vaccination negatively interfered with immunogenicity. [16] Surender Khurana et al. [17] indicated that repeated vaccination negatively affected antibody binding, antibody affinity maturation, and hemagglutination inhibition responses to H1N1, H3N2, and B strains manufactured by three different vaccine platforms. At the same time, results showed that repeated vaccination failed to induce CD4 T cell activation. [7] Studies demonstrated that prior-year vaccination correlated with low production of antibody-secreting cells and reduced effector B-cell responses to new vaccine immunization. [18, 19] The concept of antigenic imprinting has been suggested to explain the negative effect of repeated vaccination. [20] When contacted with a novel virus strain similar to an individual exposed, the immune response to the original one was predominantly boosted at the expense of response to the subsequent one. These participants’ immune systems tended to induce a strong anamnestic response to the original strains, and the production of their memory cells stimulated by the subsequent strains was reduced.

Health conditions were important factors influencing the body’s immunity. [6] In this study, participants with chronic diseases had a higher risk of lower seroconversion rates. A similar study demonstrated that hypertensive subjects had lower antibody levels against the COVID-19 mRNA vaccine. [21] A recent study revealed that this association might result from a dysfunctional immune system. [22] A study on antibody responses following vaccination with adjuvanted influenza vaccine in immunocompromised children (including AIDS, congenital immunodeficiency, and autoimmune diseases) showed that their geometric mean titer (GMT) and seroprotection rates were lower compared with immunocompetent children. [23] This phenomenon could be due to the defects in their immune system, causing worse responses to the influenza vaccine. [24] However, diabetes status seemed to have less impact on the immunogenicity of the influenza vaccine. Sarah Spencer et al. [25] found that the serologic response to the influenza vaccine was similar in participants with and without type 2 diabetes mellitus. Daniela Frasca et al. [26] found that type 2 diabetes patients had normal in vivo and in vitro B cell responses to the influenza vaccine. The possible reasons for this were that the increase in their serum LPS and sCD14 would stimulate B cells, and the TLR4 expression level in their B cells also increased. [26] Therefore, the chronic underlying medical conditions affecting the immune response to the influenza vaccine were complex. These implied that vaccine boosters or different vaccine schedules should be used for patients with chronic diseases to increase vaccination effectiveness.

Participants of different ages also differed in immune responses to the influenza vaccine. In the current study, participants aged 18 to 65 had higher fold changes and seroconversion rates, and this was consistent with previous studies. A Polish study evaluating the influenza virus circulation in the 2015/2016 epidemic season reported that both GMT levels and seroprotection rates of participants aged 0 to 4 years and older than 65 years were lower, [27] suggesting the susceptibility of these two age groups to influenza virus. The immune system of infants is not completely developed, and the magnitude and activity of their antigen-presenting cells (APCs), immune cells, and cytokines are lower than in older children and adults. [28] For older people, Goodwin et al. [29] conducted a quantitative review of studies concerning antibody levels for the influenza vaccine. They reported that the elderly over 65 had significantly lower antibody levels than younger adults, as well as the seroconversion rate, seroprotection rate, and the GMT level. However, the underlying reason for the worse responsiveness to vaccines in the elderly was “immunosenescence”, referred to as age-related changes in the immune system. [30] Aging negatively affects the body’s immune cell repertoire and results in cell-intrinsic defects in lymphocytes. [30] These contribute to the deterioration of innate and adaptive immune responses. [31] Influenza vaccines can provide moderate protection against viruses, but such protection is reduced in younger children and the elderly, and these two groups of people become vulnerable. Optimized vaccination strategy and new vaccines with improved immunogenicity were needed to reduce influenza-related morbidity and mortality.

Intriguingly, there seemed to be a trend for A/H3N2 that the higher the frequency of common colds, the better the responsiveness. This was observed in both multivariable logistic regression analysis and the rank-sum test. We speculated that the common cold could generate cross-reactive binding antibodies against the influenza vaccine strain, [32] similar to Sealy’s study [33] et al. The study indicated that the pre-existing immunity conferred by an individual’s past exposures to common cold human coronaviruses might influence the body’s control for SARS-CoV-2. [33] The common cold is caused by well-adapted pathogens and is less harmful to their hosts. [34] Therefore, people who often catch a cold have relatively low immunity and are also vulnerable to the influenza virus. Influenza vaccination is necessary for this group of people, who can benefit more from it. However, more research is needed to explore the association between the common cold and the immune response to the influenza vaccines from various aspects, providing more implications for vaccination.

Gender is an important factor affecting the immunogenicity of influenza vaccines. [35] Engler et al. found that women had significantly higher GMT than men in the case of different doses and strains. [36] However, in the current study, only the antibody fold change for B/Victoria showed a significant difference between the male and female participants. In fact, the effect of gender on vaccine immunity is complex. Besides genetic factors, the difference can also be explained through the divergent levels of sex steroid hormones, which are changing with aging. [35] [37] Women seem to lose their immunological advantage after menopause. [35] Therefore, the composition and dosage of influenza vaccines need to be adjusted according to the sex-mediated immunity differences. Future studies need to explore more factors that mediate sex differences in the immune responses deeply and extensively.

This study identified several factors relating to responsiveness to the influenza vaccine and suggested the need for more optimized vaccination strategies for susceptible groups to improve the efficacy of influenza vaccines, which is of great importance for public health. The findings in this study were a supplement to previous studies and may give some new insights into the immunogenicity response to the vaccine. However, there were some limitations in the current study. This study was descriptive, with some participants’ characteristics not collected comprehensively, and we did not perform mechanism studies based on the study cohort. The association between the immune response to different strains and host factors should be certain in the future. It is also necessary to perform larger cohort studies and experiments to explore the underlying mechanisms.

The updated position paper on the use of seasonal influenza vaccines published by WHO recommended that older adults, children, health workers, pregnant women, and individuals with comorbidities and underlying conditions be prioritized as target groups. [38] In the future, it is of significance to increase vaccination rates for these vulnerable populations and optimize vaccine components and vaccination strategies to improve vaccine efficacy and reduce the morbidity, mortality, and disease burden associated with influenza.

## Conclusions

This study explored the factors associated with the immune response to influenza vaccines in China. The results showed that repeated vaccination, health condition, age, and frequency of colds were important factors affecting the immunogenicity responses to influenza vaccines. The results may provide supporting data for identifying low responders and optimizing the vaccination strategies to improve the effectiveness of influenza vaccines in human.

## Data Availability

The datasets generated and analysed during the curret study are not publicly available due to existing general data protection rules and official secrecy, but are available from the corresponding author on reasonable request.

## **Abbreviations**

OR: Odds Ratio
CI: Confidence Interval
CDC: Center for Disease Control
TIV: Trivalent Inactivated Vaccine
QIV: Quadrivalent Inactivated Vaccine
WHO: World Health Organization
HA: Hemagglutination
HAI: Hemagglutination Inhibition
SPF: Specified Pathogen-Free
RDE: Receptor Destroying Enzyme
BMI: body mass index
GMT: Geometric Mean Titer
P_25_: Lower quartile
P_75_: Upper quartile
